# Relatives’ experiences of an equine-assisted intervention for people with psychotic disorders

**DOI:** 10.1080/17482631.2022.2087276

**Published:** 2022-06-13

**Authors:** Linda Fridén, Sally Hultsjö, Marie Lydell, Henrika Jormfeldt

**Affiliations:** a School of Health and Welfare, Halmstad University, Halmstad, Sweden; b Department of Psychiatry, Region Jönköping County, Sweden and Department of Health, Medicine and Caring Sciences, Division of Nursing and Reproductive Health, Linköping University, Linköping, Sweden

**Keywords:** Equine-assisted interventions, experiences, health capabilities, mental health, psychotic disorders, qualitative content analysis, relatives

## Abstract

**Purpose:**

The aim of this study was to describe relatives’ experiences of an equine-assisted intervention for people with psychotic disorders.

**Methods:**

The study has a qualitative and descriptive design. Ten semi-structured interviews were performed with relatives of people with a psychotic disorder who had participated in an equine-assisted intervention. A conventional content analysis was used to analyse the data.

**Result:**

The overall category “Being with the horses strengthens health capabilities” summarizes the four identified subcategories “The horses contribute to a context with a common focus”, “Interaction with the horses enhances self-confidence and motivation”, “The interplay with the horses nurtures positive emotions” and “Being with the horses encourages physical activity”.

**Conclusions:**

The result of this study contributes to the growing body of research about the potential outcomes of equine-assisted interventions. The result also indicates that equine-assisted interventions may improve health capabilities among people with psychotic disorders. The generated knowledge may be useful in mental health services when developing equine-assisted interventions.

## Introduction

Approximately 20 million people worldwide have been diagnosed with the psychotic disorder, schizophrenia, between 1990 and 2017 (Abate et al., 2018). Symptoms associated with psychotic disorders can be divided into positive and negative dimensions. Positive symptoms include delusions, hallucinations and disorganized thinking whereas the negative symptoms include reduction in expression of emotions, poverty of content of speech, lack of motivation and reduction in the ability to feel anticipation and joy (Maj et al., 2021). Psychotic disorders are often associated with experiences of stigmatization and discrimination in close relationships, social contexts and healthcare (Brain et al., 2014). This may lead to unrealistic interpretations of one’s own and others’ emotions and also social isolation (Csukly et al., 2013). Feelings of loneliness, isolation, hopelessness, as well as a lack of belonging, may lead to an increased risk of suicide (Barut et al., 2016). A lack of belonging to social contexts may negatively affect the quality of life of people with psychotic disorders and lead to depression (Siegrist et al., 2015). A link between cognitive difficulties and a lack of social contexts, passivity and inactivity has been recognized (Fleury et al., 2013). Physical activity has shown to be an important prerequisite for increased self-confidence and reduced psychiatric symptoms in people with mental illness (Erdner & Magnusson, 2012) and physical inactivity has shown to be a risk factor for physical ill-health such as the metabolic syndrome (Nyeboe & Lund, 2013).

Healthy living, in terms of daily structures with sufficient sleep, motivational life events and supportive significant others, has been described as important among people with psychotic conditions (Blomqvist et al., 2018; Schnor et al., 2021). According to Tengland (2020) health is related to Nussbaum’s theory (Nussbaum, 2011) of ten different capabilities that are essential to live a full life. Focusing on abilities when caring for people with psychotic disorders has been described as beneficial (Verhaeghe et al., 2013) and interventions should be adapted to the patient’s current ability and state of health (Happell et al., 2012). However, the daily care provided for people with psychotic disorders often lacks the necessary support in terms of healthy routines and meaningful life content (Jormfeldt & Hallén, 2016). As this group, due to lack of motivation, are less likely to participate in health-promoting interventions (Holt et al., 2018; Speyer et al., 2016; Vancampfort et al., 2011), interventions to support lifestyle interventions are even more important (Erdner & Magnusson, 2012; Holt et al., 2018; Speyer et al., 2016). Further research regarding how to successfully support and encourage physical activity among people with psychotic conditions is needed (Holt et al., 2018; Speyer et al., 2016).

Equine-assisted interventions, EAI, is an umbrella term for all types of interventions where horses are an important part of the therapeutic process (Lee et al., 2016). EAI does not rely solely on verbal language as many other traditional therapies do (Wilson et al., 2017) and they require a certified leader who helps guide and interpret the horse’s behaviour (White‐Lewis, 2020). EAI has shown the potential to contribute to increased self-confidence and improved ability to communicate with other people (Wilson et al., 2017) and have also shown an increase in positive emotions and a decrease in negative emotions (Schütz & Schmitz, 2021). EAI, in which horses can function as therapeutic facilitators, has shown biopsychosocial benefits and therapeutic outcomes in several types of mental illnesses (Boss, 2019; Kern-Godal et al., 2016b; Lee et al., 2016; Nurenberg et al., 2015 & Wilson et al., 2017). The horse’s behaviour reflects the person’s energy without causing any judgemental feelings (Wilson et al., 2017) and research has shown that symptoms of the disease can be reduced more rapidly when animals are involved in the therapeutic process in the treatment of people with psychotic disorders, depression, phobias, substance abuse problems (Dimitrijevic, 2009) and post-traumatic stress disorder (Boss, 2019). People with psychotic disorders have previously described that being with the horses in EAI evoked healing powers, had a calming effect, made them feel normal and important as well as contributed to an awareness of their own and others’ behaviour (Hultsjö & Jormfeldt, 2021). Being with the horses was also found to help the intervention participants to develop leadership qualities, feel pride and feel more harmonious. Other positive outcomes of EAI for people with psychotic disorders have been noted, such as increased confidence and self-esteem, improved social skills, enhanced enjoyment as well as increased activity (Jormfeldt & Carlsson, 2018). A lack of knowledge about EAI in society and the healthcare services constitutes a serious obstacle for this therapy to become more accepted and become an officially adopted part of the range of care provided (Wilson et al., 2017). There is also a need for more high-quality studies regarding possible outcomes of EAI for people diagnosed with psychotic disorders (Anestis et al., 2014; Kendall et al., 2015; O’Haire, 2013; Stern & Chur-hansen, 2019).

Relatives often spend a large amount of time caring for their diagnosed family members (Brain et al., 2018; Flyckt et al., 2013). Relatives’ experiences are an important source of knowledge (Zegwaard et al., 2015) and mental health services should be based on a holistic view of both the patient and their relatives (Svensson et al., 2020). Interventions that allow relatives to be involved may also improve social functioning and the emotional ability of people with a psychotic disorder (McFarlane, 2016 & Pharoah et al., 2010). Furthermore, this group often has difficulties making their voice heard due to their symptoms (Maj et al., 2021) and their relatives have an important task in mediating their opinions and wishes since the relatives often have a broad insight into their family member’s daily lives (Olasoji et al., 2017) and know what their capabilities are. The relatives can therefore contribute with valuable knowledge when studying their experiences of EAI and to our knowledge, there are no previous studies addressing relatives’ experiences of EAI for people with psychotic disorders. Relatives’ experiences of health promotion interventions for their family members with a psychotic disorder can contribute to greater knowledge in order to improve the health of this group. The aim of this study was therefore to describe relatives’ experiences of an equine-assisted intervention for people with psychotic disorders.

## Method

### Design

The study had a qualitative descriptive design based on semi-structured interviews with relatives of people diagnosed with a psychotic disorder who participated in an EAI. An inductive qualitative conventional content analysis was used to capture the relatives’ narratives and experiences (Hsieh & Shannon, 2005).

### Participants

The inclusion criteria for participation in the study were: to be over 18 years of age and related to or having a close relationship with a person with a psychotic disorder who had participated in the EAI described below. The participants in the EAI were recruited through a local user association for people with schizophrenia or schizophrenia-like conditions at a members’ meeting and the interviewed relatives were recruited through the participants in the EAI. The project manager gave oral and written information about the study when recruiting them. Further information was provided by the interviewers to the individuals who consented to participate in connection with the interviews one to six months after the intervention was complete. Finally, twelve relatives were approached and ten of them agreed to participate in the study. The sample consisted of eight women and two men aged between 26 and 82 years, and included five mothers, one father, one daughter, one sister, one brother-in-law and one close friend. Six of the ten interviewed relatives used the opportunity to accompany their family members to at least one of the EAI occasions.

### The equine-assisted intervention

An EAI was offered to people with psychotic disorders to support holistic health.

The EAI consisted of a 12-week group intervention performed six times, once every 14 days.

The family members of the interviewed relatives were all diagnosed with schizophrenia or a schizophrenia-like condition and they were between 32 and 57 years of age, equally divided between men and women. The groups of five to ten participants met every fortnight for a minimum of six occasions during the period of August 2018 to December 2020. The groups were led by a psychiatric nurse who was certified to conduct EAI by the Swedish National Organization for Equine Assisted Interventions (OHI) and is from now on referred to as an OHI-certified therapist. The intervention started and ended with a joint 30 kilometres journey back and forth to the horse farm and the urban area. Each session lasted four hours and included a short introductory gathering where the participants planned the activities of the day, most often a one hour walk in the rural landscape with the horses. To ensure the safety of both participants and horses there was one handler for each horse available during the walks. The activities were conducted followed by a meal and a concluding gathering with reflective dialogue about feelings and needs generated during the activities with the horses and how these feelings and needs could be related to the participants’ lives in general. The reflective dialogue was performed with the aid of non-violent communication cards (Rosenberg, 2015). The activities together with the horses were adapted to the needs of each individual participant. For in-depth details about the intervention see, Hultsjö and Jormfeldt (2021).

### Data collection

The ten interviews were conducted by registered nurses with experience of conversations with patients and relatives between November 2019 and February 2020. A semi-structured interview guide was used consisting of open questions and follow-up questions were continuously asked to allow the interviewed relatives to develop their answers as well as be given time and opportunity to express themselves freely on the subject (Hsieh & Shannon, 2005). At the request of the relatives, three of the interviews took place at the interviewed relatives’ homes, one interview took place at a library and one interview was conducted via digital video meeting. The last five interviews were performed by telephone since face-to-face interviews were not an option due to the covid-19 pandemic. Each interview started with an opening question about how the interviewed relatives would like to describe the EAI that their family members have participated in and in what way they feel that this may have contributed to their health and if so in what way. The interviewed relatives were then asked to reflect freely upon their experiences of their family members’ participation in the EAI. The interviews lasted between 12 and 45 minutes, with a median of 23 minutes and they were audio-recorded and transcribed verbatim.

### Data analysis

Qualitative conventional content analysis according to Hsieh and Shannon (2005) was used to analyse the interviews. First, the interviews were read repeatedly to acquire an understanding and a sense of the whole. In the next step, data was read word by word and data related to the aim of this study were identified and coded to describe key thoughts. The codes with similar content were reflected upon and placed in subcategories and one hierarchically superior category emerged, the overall category (see Table 1). The process was not linear as it went back and forth as the authors discussed the material to find an agreement about how to most accurately code and categorize the content. The attempt was to find descriptions close to the text, even though some interpretation was required (Hsieh & Shannon, 2005). The analysis was discussed among the co-authors on several occasions and led to a regrouping of the codes and subcategories.

**Table 1.** Examples of meaning units, condensed meaning units, codes, subcategories, and the overall category

Meaning unit	Condensed meaning unit	Code	Sub-category	Overall category
*“Partly, I believe that in the whole that it’s being needed, that they have a day they are supposed to be there, that someone is expecting them, and partly the group, that it’s something you do together, you are a part of a bigger context”*(Interview 6)	To be needed, expected and have something you do together in a group makes you a part in a larger context	Participating in a meaningful social context	The horses contribute to a context with a common focus	Being with the horses strengthens health capabilities
*“Oh, maybe they feel why should I walk alone, but when the horse also walks, they may feel that we can exercise together”* (Interview 1)	Walking alone has no meaning, but when the horse walks, we are exercising together	The horse gives exercise meaning	Being with the horses encourages physical activity	
*”… she’s become more positive…. when she meets resistance, she takes it a bit easier. A bit easier than she did before perhaps… Still, she knows she’s loved (by the horses) for who she is and she’s got a better self-confidence”* (Interview 5).	Has become more positive, takes resistance easier. Feels like she is loved for who she is and has better self-confidence	More positive, copes with resistance better, feels unconditional love and has better self-confidence	Interaction with the horses enhances self-confidence and motivation	
*“The horse and nature, it’s the interplay that’s a bit like a happy pill”*(Interview 1)	The horse and nature have an interplay that is like a happy pill	The interplay of horses and nature is a happy pill	To be in the presence of the horses nurtures positive emotions	

### Ethical considerations

The study was approved by the Regional Ethical Review Board, Lund University, Sweden, Dnr 2017/709.

The participants were given written and oral information about the purpose and the structure of the study several months before the interviews, and they gave their written informed consent in conjunction with each interview. Information was given that participation was voluntary, that participants could withdraw from the study at any time without having to express any reasons and that the interviews were treated with confidentiality. The study was performed according to the regulations of the World Medical Association Declaration of Helsinki (World Medical Association Declaration of Helsinki, 2013).

## Result

The analysis of the data generated the overall category: “*Being with the horses strengthens health capabilities*”. This overall category was formed by four subcategories “*The horses contribute to a context with a common focus*”, “*Interaction with the horses enhances motivation and self-confidence”, “The interplay with the horses nurtures positive emotions”, and* “*Being with the horses encourages physical activity*”.

### Being with the horses strengthens health capabilities

The overall category “Being with the horses strengthens health capabilities” was formed by the four subcategories as being with the horses seemed to nurture positive emotions and reduced malaise and sedentary behaviour, which therefore promoted the health of the family members, according to their interviewed relatives. The interaction with the horses was described both as something natural and relaxing and as a process of growth, from being at a secure distance to developing the courage to come closer to and interact with the horse. Positive emotions were experienced in the horses’ presence and the interviewed relatives conveyed that their family members looked forward to the equine-assisted activities with an expectant tingling in the stomach and joy before the activity. The horses were perceived as beautiful and the closeness to the horses led to joy and relaxation among the family members. The interviewed relatives also noticed that their family members were happy when the horses came forward and greeted them. It was also stated by the interviewed relatives that their family members experienced love when being with the horses. Furthermore, the interplay between horse, nature and man was described as a “happy pill” and the horses were perceived by the interviewed relatives as generating feelings of belonging and a feeling of being welcome among their family members.

#### The horses contribute to a context with a common focus

“The horses contribute to a context with a common focus” formed the first subcategory as the interviewed relatives defined the EAI in a group with other participants as a meaningful context for the intervention. The interviews highlighted the problem of a lack of available activities and difficulties in getting the family members involved in, for example, family activities. Since the interviewed relatives had the opportunity to accompany their family members to the EAI, they experienced that it became an opportunity to deepen their relationship. They shared the experience with the horses which they could remember and relate to together.

… to be able to participate, be there with X, see X laugh and make a small jump, you share something that isn’t just everyday activities, but something new and exciting that we can talk about (Interview 6).

Several of the interviewed relatives described loneliness as a problem for their family members and therefore emphasized the importance of them being given the opportunity of belonging in a context, which did not primarily focus on them as someone with a disease or being different.

It’s much about being outdoors, that he comes out, that it’s a group, that he’s involved … (Interview 1).

The interviewed relatives described how the horses contributed to a context, the horses were an important focal point, towards which everyone’s eyes were turned. The interviews showed how the family members, despite their differences, gathered around the horses as a functioning unit. The horses constituted a crucial precondition for the family members’ active involvement, both in the interaction with the horse and with the other participants.

… they all have different symptoms and diagnoses, quite different diagnoses, but it was like a group there, gathering around and functioning as a unit, irrespective of what it looked like (Interview 4).

The family members were given the opportunity to actively decide, for example, which horse they wanted to interact with or whether they wanted to groom or walk the horse in the woods, which the interviewed relatives described as particularly positive. The family members also developed skills in interacting with the horses and participated in the social gathering in the context characterized by the horses and the horses’ environment.

Nobody feels, nobody should feel forced, but it should become clear what the person wants and feels that he/she dares to do (Interview 9).

The horses provided the family members with a reason to express their thoughts and opinions about life in general during the joint activity with the horses. The interviewed relatives described the fellowship with the horses as a natural reason to express thoughts, which was described as something that was often lacking in other contexts. There was also a desire among the interviewed relatives that the EAI should take place more often and they also expressed a wish that their family members could participate in more contexts where their abilities could be further developed.

Partly, I believe that in the whole that it’s being needed, that they have a day they are supposed to be there, that someone is expecting them, and partly the group, that it’s something you do together, you are a part of a bigger context (Interview 6).

#### Interaction with the horses enhances self-confidence and motivation

In the second subcategory “Interaction with the horses enhances self-confidence and motivation” the interviewed relatives revealed experiences of becoming aware of their family members’ increased motivation, which was generated during the activities with the horses. The interviewed relatives portrayed the horse-assisted interventions as a new experience for their family members. The former also spoke of their family members usually having difficulties getting started with activities. They also added that people with psychotic disorders are not prioritized in the range of activities provided by the municipalities and are not given sufficient support to be able to benefit from or participate in the available range of activities. The interviewed relatives revealed that people with psychotic disorders often lack inspiring activities, but the interviewed relatives had noticed that their family members were motivated in an entirely new way when participating in the activities together with the horses.

It emerged in the interaction with the horses, that the family members displayed abilities, that the interviewed relatives did not know that they had. The interviewed relatives also pointed out that their family members developed skills during the activities such as the courage to lead the horses and get them to cooperate.

… she’s become more positive … . when she meets resistance, she takes it a bit easier. A bit easier than she did before perhaps … Still, she knows she’s loved (by the horses) for who she is and she’s got a better self-confidence (Interview 5).

The interviewed relatives revealed that the family members grew with the task and gained increased self-confidence and self-esteem and they also seemed to gain a sense of pride in their accomplishments.

They’ve been walking at the same pace as the participants’ pace and it’s important that they feel pride, that they can handle an animal this big, that’s a feeling of security. It’s probably good for their self-esteem as well (Interview 10).

The interviews gave examples of how the family members interacted with the horses on their terms, as they could participate in the activities based on their own will and ability. The interviewed relatives also emphasized the positive aspect of something, which managed to capture the attention of their family members.

You walk with the horse and it’s quite impressive, it’s like a challenge, but he goes, he meets that challenge, he gets comfortable (Interview 7).

It was described that the horses required full focus during the activity so that they would not walk away, or do as they pleased, unlike smaller animals, which were perceived as requiring less effort to control and therefore might not have had the same therapeutic effect. The interviewed relatives spoke of it being particularly remarkable that their family members wanted to participate in the activities with the horses despite the cold and rainy weather, which was experienced by the interviewed relatives as something new, as they had previously often refrained from participating in activities if there were any undesirable circumstances connected to the activity. The horses helped the family members get out of their comfort zones and enhanced their abilities to function in a social context according to their interviewed relatives.

Although it’s bad weather you go there, the horse is a driving force. Otherwise, if the weather is half-bad you don’t go out. But then it’s a goal that there’s someone waiting for you, yes they go there, walk and they get to lead the horse. (Interview 10).

#### The interplay with the horses nurtures positive emotions

In the third subcategory, “the interplay with the horses nurtures positive emotions”, the interviewed relatives described various ways that being with the horses affected their family members’ emotions positively prior to, during or after attending the EAI. The interviews conveyed that the intervention raised the mood of the family members, and they were enjoying the time spent with the horses. Prior to starting the EAI they felt positive anticipation, during the EAI they felt joy and happiness and afterwards, the interviewed relatives noticed that the family members shared stories about the good times they had experienced with the horses. One interviewee also said that a participant in the invention also had experienced the joy of feeling loved in the interaction with the horses.

The horse and the nature, it’s the interplay that’s a bit like a happy pill (Interview 1).

None of the interviewed relatives mentioned any negative emotions associated with the EAI, and if the family members would need a moment alone if the social interaction was too difficult, they experienced that there was always space and opportunity for some alone time and then be able to return to the group in a better mood.

Encountering and being together with another living being has also been described by the interviewed relatives as generating a longing to be a part of the warmth, power and life that radiates from the horse. The interviewed relatives noticed that it seemed special to be in the presence of the horse and their family members seemed to get the feeling that they have an important task in caring for and walking with the horse.

He probably has a longing to somehow meet the horse, that it’s an animal that radiates warmth and power and life and that it was, and that he, X, then gets to be a part of it. He’s sort of allowed to be in the presence of the horse (Interview 6).

The interviewed relatives described different ways that their family members experienced calm linked to the EAI. It was described that horses make people around them calmer and that they reduce anxiety and that horses are wise animals that can detect a little of what the human is feeling and that those attributes contribute to calm and relaxation in their family members.

The interviewed relatives revealed that both the horse and the OHI-certified therapist were important as they made their family members feel welcome. The horse was perceived as a powerful animal and it was necessary to be present in the interaction, which according to one of the interviewed relatives could lead to the family members having to let go of their focus on the psychotic symptoms, such as hearing voices.

One thing is sure with the group that is a plus for him, they feel that they are a part of the society instead of being an outsider (Interview 7).

The interviewed relatives expressed that the way that the OHI-certified therapist, who was responsible for the intervention, approached the family members also was of importance. The OHI-certified therapist was said to have a sensibility for individual needs, a competence to understand the mental illness issues and an ability to treat the family members with respect and as normal persons of equal value, which contributed to the positive effects according to the interviewed relatives. The OHI certified therapist’s competence regarding both the horse and mental health issues were perceived as important for guiding the family members in safely connecting with the horses that at the same time felt genuine and natural in the context around the horses. Some of the family members had previously stopped participating in activities because they felt uncomfortable or did not feel welcome there, but the activity with the horses was an activity they did not want to miss out on despite obstacles such as rainy weather. One of the interviewed relatives expressed that it was the interplay between both the horses and the people responsible for the activity that made the family member so fond of attending the EAI.

If the staff had been awkward, it wouldn’t have been as appealing, despite the presence of the horses. So, all the pieces of the puzzle are in place with the staff and the way they received her and that she felt welcome and allowed to be herself (Interview 10).

#### Being with the horses encourages physical activity

“Being with the horses encourages physical activity” formed the fourth subcategory as interviewed relatives spoke of their family members increasing their level of physical activity during the interaction with the horses. The interviewed relatives also talked of their family members having both the motivation and the ability to be physically active when they interacted with the horses. The importance of the horses to encourage physical activity was emphasized by the interviewed relatives and they revealed that their family members had previously had difficulties attending various physical activities but stated that the EAI made them more physically active than in other contexts without horses.

It was good with the horses that she got out and got exercise. She usually doesn’t do any physical activity (Interview 4).

The interviewed relatives described that their family members felt that it was meaningful to walk with the horses as they appreciated the experience of nature and thought that the forest, as well as the horses, were beautiful.

Maybe they feel, why should I walk alone? But when the horse also walks: now we exercise. They do it together (Interview 1).

The interviewed relatives emphasized the importance of the physical activity to prevent physical disease and reduce obesity and diabetes. The family members were described as physically active together with the horses and they also increased their physical activity between the intervention occasions and after it was completed. One example of this increased activity was that one of the family members started to walk regularly and take longer walks than he had done prior to taking part in the intervention. The interaction with the horses was perceived by the interviewed relatives as being a motivation for physical activity. To meet the horse was the goal and the exercise came along as a bonus as the encounter with the horses made the physical work worth the effort.

She doesn’t engage in any physical activity otherwise, this has been something special, different from everything else and she was looking forward to it (Interview 5).

## Discussion

### Methodological considerations

The approach with a conventional content analysis is considered appropriate when very few previous studies have been carried out in the research field (Hsieh & Shannon, 2005). Although the sample was purposeful and relatively small, the interviewed relatives represented a variety of gender and type of relation to the participant in the intervention and therefore constituted a diverse sample. The fact that the interviews were performed by different authors, that some of the interviews were made by telephone and that one of them was relatively short may have affected the quality of the data from the interviews. Despite this, all the interviews contributed with valuable knowledge. Telephone interviews may have some advantages as the interviewees may feel more comfortable and relaxed than in face-to-face interviews (Ward et al., 2015). Saturation can be said to have been achieved since the content of the final interviews was mostly consistent with the earlier interviews. Furthermore, being as the aim of the study was descriptive a smaller sample could therefore be considered sufficient (Hennink et al., 2017). Researcher triangulation (FitzPatrick, 2019; Hsieh & Shannon, 2005) was used as all the authors discussed and determined codes and categories together until consensus was attained in order to promote trustworthiness. Limitations to this study were that the intervention only included a minimum of six occasions and that there were two weeks between the sessions.

### Discussion of results

The main results of this study show that the interviewed relatives described the horses as contributing to a context with a common focus when their family members were gathering around the horses and they also experienced that the horses in the EAI encouraged physical activity. Furthermore, the interviewed relatives described the interaction with the horses as enhancing self-confidence and motivation as well as nurturing positive emotions among their family members.

The relatives’ perspective has previously been recognized as important in the care of people with schizophrenia (Wainwright et al., 2014). Hultsjö and Jormfeldt (2021) performed interviews with the participants in the same intervention, with the aim of exploring the role of the horse in the EAI as seen by people with psychotic disorders. Various similarities, for example, being calmer and more relaxed, were found between the latter study and the results of the present study, which strengthens the assumption that the relatives have a broad knowledge about their family members’ needs that are required to be met in a health promotion intervention.

The horses contributed to a context with a common focus for the participants in the EAI from the interviewed relatives’ perspective. The horses formed a focal point and despite the differences between the participants, they met around the horses as a functioning unit. Barut et al. (2016) also describe the importance for people with psychotic disorders of belonging in a context, as it inspires hope and increases treatment adherence and contributes to recovery. Belonging in a context was also seen by Svedberg et al. (2004) as important when it comes to experiencing health in people with psychotic disorders. The results also show that the interviewed relatives had concerns about the loneliness felt by their family members and highlighted the importance of allowing them to belong in a context. The relevance of this concern has been confirmed by Juncheng and Jie (2014) who showed that social isolation is a risk factor for deteriorating health conditions among people with psychotic disorders. The participants in the EAI received the possibility of improving their capability of being able to affiliate with other people (Nussbaum, 2011). They all had unique needs and prerequisites in the EAI context, but the horses gave them a reason to socially interact as a group and in doing so they create a relationship with each other, the OHI-certified therapist as well as the horses.

Interaction with the horses enhances self-confidence and motivation since the interviewed relatives experienced that their family members with a psychotic disorder were motivated to participate in the EAI due to the horse’s presence. Further, the interviewed relatives perceived that this interaction with the horses contributed to increased self-confidence among their family members during their participation in the intervention. Previous research has shown that people with permanent mental illness are described as being difficult to motivate (Happell et al., 2012). The interviewed relatives spoke of their family members previously lacking health-promoting initiatives and that the range of health-promoting activities was insufficient. They also described that the support for participating in activities was insufficient. A lack of initiative and social difficulties can be a problem when living with a psychotic disorder and it may lead to difficulties in being a part of social activities and other health-promoting activities (Maj et al., 2021). The perceptions of the interviewed relatives that the EAI was an activity that their family members enjoyed, looked forward to and did not want to miss out on is, therefore, an important aspect. This indicates that interventions that can help increase motivation are important to be able to stimulate people with psychotic disorders to participate in interventions aiming to promote health.

The horses are highlighted in the results as something that required full attention and active participation. The interaction with the horses and the other participants in the EAI corresponds to Nussbaum’s capability of using practical reasoning (Nussbaum, 2011). The participant must be alert and make decisions about what to do next when managing the horse. Furthermore, practical reasoning is needed to assess the situation and to try various ways of getting the horse to cooperate. The participants’ self-confidence and motivation may increase when the horse cooperates, which in turn could result in strengthened health capabilities.

The result also revealed that the interplay with the horses nurtures positive emotions and in doing so the EAI has the potential to promote health since positive expectation and joy are important building blocks for quality of life and important aspects of health and recovery for people with psychotic disorders (Boyer et al., 2013). The EAI has the potential to increase several of Nussbaum’s (2011) capabilities, such as being able to use one’s mind, imagination and thought, being able to have emotions and care for and have relationships with others and being able to play, laugh and enjoy recreational activities. Experiencing positive emotions when interacting with the horses has previously been shown to be associated with EAI in a number of studies (Hultsjö & Jormfeldt, 2021; Kern-Godal et al., 2016a; Pohl et al., 2018; Schütz & Schmitz, 2021)

The relatives experienced that their family members became calmer and more relaxed when being around the horses. This has also been described by the intervention participants themselves in the study by Hultsjö and Jormfeldt (2021). Horses have a slightly higher body temperature and a slightly lower respiratory rate than humans, which means that close body contact with horses is often perceived as warming and calming (Kern-Godal et al., 2016b; Lee et al., 2016; Nurenberg et al., 2015; Wilson et al., 2017).

The findings of this study revealed that the EAI constituted a meaningful activity where the participants felt welcome, normal and belonging in a context. The results also emphasize the importance of feeling normal and not being treated differently because of a mental health disorder in order to be able to appreciate participating in an activity. This is consistent with Barut et al. (2016) who found that the sense of belonging made life easier and more joyful and being a part of something made life more meaningful for people with psychotic disorders. Hultsjö and Jormfeldt (2021) also described that the participants in the EAI experienced that being with the horses made them feel normal and that they could more easily cope with their mental health condition and even forget about their symptoms when being with the horses. The capability of being able to control one’s environment (Nussbaum, 2011) may in the EAI context be seen as a need to have the possibility of influencing the activities that one takes part in. The actual intervention was perceived as involving a large degree of self-determination that was described as positively affecting the participants. Self-determination was described as a key factor in the EAI and previous research has shown that different individuals may benefit from different parts of an EAI. These findings indicate that an individualistic approach is especially important (Carlsson, 2017).

The level of physical activity among the participants was increased during the intervention according to their interviewed relatives. Although, one difference between the present study and that of Hultsjö and Jormfeldt (2021) was that the relatives emphasized physical activity as an important outcome of the EAI due to the positive physical effects, whilst this was not pronounced as the main focus by the participants in the intervention themselves (Hultsjö & Jormfeldt, 2021). This corresponds with the interviewed relatives’ descriptions of the horses being the reason for participating in the EAI, and where the physical activity is not the primary motivation but came instead as a bonus. Physical activity has previously shown a positive effect on self-confidence, creating meaning and reducing anxiety and worry in people with psychotic disorders (Van Meijel et al., 2009) as well as increased hope for the future (Kukla et al., 2013). The interviewed relatives emphasized that the horse gave meaning to the physical activity during the EAI for their family members so that they were able to naturally appreciate being in nature with the horses and therefore automatically moved more. Since it has been shown that a lack of physical activity is common among people with psychotic disorders (Lundström et al., 2019 & Manu et al., 2015) and that the main focus of the mental health services is often on the psychotic disorder and not the physical health (Happell et al., 2012), it can therefore be valuable to find motivational interventions that also encourage bodily movement. The increase in physical activity as an effect of the EAI as seen in the result can contribute to enhancing the capabilities to live a full lifespan and have physical health (Nussbaum, 2011).

### Conclusion and implication

The result of this study shows the relatives’ perspective that participation in the EAI to strengthen health capabilities among their family members diagnosed with a psychotic disorder. The findings indicate that horses contribute to a context with a common focus, nurture positive emotions and enhance self-confidence and motivation as well as encourage physical activity among the intervention participants. Another finding of this study is that relatives can be able to contribute with important complementary information about the benefits of participating in the EAI for their family members. Relatives emphasize that the EAI encourages physical activity among their family members, which was not emphasized by the intervention participants themselves in a previous study by Hultsjö and Jormfeldt (2021). Overall, the results indicate that EAI has the potential to enhance several health capabilities, such as being able to use one’s mind, imagination and thought, harbour emotions, care for others and being able to enjoy recreational activities among participants with psychotic disorders. To feel welcome and belonging in a healthy normal context seems extremely important for the intervention participants to be able to appreciate participating in the EAI, which puts emphasis on the importance of individual adaptation in the intervention. Further investigations should focus on how individually adapted EAI could be integrated into the existing healthcare system to improve health capabilities and in turn contribute to overall health and decreased stigmatization among people with psychotic disorders. Further research is needed to provide policymakers and managers in the healthcare and social services with sufficient knowledge for them to be able to create conditions for providing EAI specifically designed for the vulnerable client group consisting of people with psychotic disorders.
